# Clinical Impact of Red Blood Cell Transfusion Location on Gastrointestinal Bleeding Outcomes: Emergency Department vs. Inpatient Unit

**DOI:** 10.3390/healthcare13141656

**Published:** 2025-07-09

**Authors:** Mehmet Toprak, Harun Yildirim, Ertan Sönmez, Murtaza Kaya, Ali Halici, Abdil Coskun, Mehmed Ulu

**Affiliations:** 1 Emergency Medicine Department, Kutahya City Hospital, 43020 Kutahya, Turkey; mehmet.toprak@ksbu.edu.tr; 2 Department of Emergency Medicine, Medical Faculty, Kutahya Health Sciences University, 37150 Kutahya, Turkey; ertan.sonmez@ksbu.edu.tr (E.S.); murtaza.kaya@ksbu.edu.tr (M.K.); ali.halici@ksbu.edu.tr (A.H.); abdil.coskun@ksbu.edu.tr (A.C.); 3 Department of Emergency Medicine, Adiyaman Research and Training Hospital, 02100 Adiyaman, Turkey; mehmed.ulu@ksbu.edu.tr

**Keywords:** gastrointestinal bleeding, emergency department, red blood cell transfusion, mortality, hospital stay

## Abstract

**Background:** Gastrointestinal (GI) bleeding is a common and potentially life-threatening condition frequently encountered in emergency departments (EDs). The optimal strategy for red blood cell suspension (RBCS) transfusion, including timing and location, remains unclear. This study aimed to evaluate the impact of transfusion location (ED vs. inpatient units) on mortality and hospital stay in patients with GI bleeding. **Methods:** A cross-sectional descriptive study was conducted in the ED of a tertiary care hospital. Patients admitted with GI bleeding between 1 June 2021, and 1 June 2023, who received RBCS transfusion were included. Data on demographics, laboratory parameters, transfusion details, and clinical outcomes were collected from the hospital information system. Logistic regression was used to identify mortality predictors. **Results:** A total of 244 patients were included. Patients transfused in the ED had a significantly shorter hospital stay compared to those transfused in inpatient units. However, mortality did not differ between the groups. Logistic regression identified age, albumin, hemoglobin, creatinine, and hospital stay as independent mortality predictors, while transfusion location was not significant. **Conclusions:** Early RBCS transfusion in the ED may reduce hospital stay but does not significantly impact mortality. Identifying mortality-associated factors is crucial for optimizing patient management. Further prospective studies are needed to clarify the role of transfusion location in GI bleeding outcomes.

## 1. Introduction

Gastrointestinal (GI) bleeding is a major cause of morbidity and mortality among patients presenting to emergency departments (EDs). Upper gastrointestinal bleeding (UGIB), particularly those originating from peptic ulcers, is associated with high mortality rates and often necessitates urgent blood transfusion. The annual incidence of UGIB ranges from 84 to 160 per 100,000 adults, with reported mortality rates between 5% and 10% [1]. In contrast, lower gastrointestinal bleeding (LGIB) typically follows a milder course; however, in elderly populations, it can result in severe outcomes [2]. Blood transfusions administered for GI bleeding aim to reduce in-hospital mortality and minimize the risk of complications, representing a crucial therapeutic approach [3].

Blood transfusion in GI bleeding is commonly performed to maintain hemodynamic stability and ensure adequate tissue perfusion. However, there is no clear consensus regarding transfusion strategies. While liberal transfusion approaches may increase the risk of mortality and rebleeding, particularly in patients with portal hypertension [4,5], restrictive strategies targeting lower hemoglobin (Hb) thresholds, generally around 7 g/dL, have been associated with better clinical outcomes [6]. A Cochrane systematic review further supports that restrictive transfusion does not increase 30-day mortality and may reduce transfusion requirements compared to liberal approaches [7]. Although such findings have shaped current recommendations, they primarily focus on transfusion thresholds rather than the context or timing of transfusion delivery.

Despite the extensive focus on transfusion thresholds, the impact of transfusion location—specifically whether red blood cell (RBC) transfusions are initiated in the ED or after hospital admission—remains poorly defined. While prehospital transfusion has been shown to be feasible and safe in selected GI bleeding cases, its impact on patient-centered outcomes remains unclear due to limited supporting data [8]. A multicenter cohort study showed increasing adherence to restrictive transfusion practices in EDs, particularly in patients with Hb levels between 7.0 and 9.9 g/dL, yet it did not assess whether transfusion location influences clinical outcomes such as mortality or hospital stay [9]. Reviews support the safety of restrictive strategies across various bleeding types but also highlight the lack of contextual analyses regarding the setting of transfusion delivery [10]. Similarly, Kerbage et al. (2024) reported no significant differences in outcomes based on Hb thresholds alone, emphasizing the importance of evaluating transfusion decisions within a broader clinical context [4]. Moreover, analyses linking commonly used risk scores—such as the Glasgow–Blatchford Score (GBS), AIMS65 (Albumin–International Normalized Ratio–Mental Status–Systolic Blood Pressure-65), and Rockall—with the timing or location of transfusion are still scarce [11].

The aim of this study was to retrospectively evaluate the impact of the transfusion site (ED vs. inpatient units) on clinical outcomes in hospitalized patients with GI bleeding who received red blood cell suspension (RBCS) transfusion. We hypothesize that rapid transfusions performed in the ED may reduce mortality; however, transitioning to inpatient care after stabilization might introduce additional risks. In this context, we compared the effects of ED versus inpatient transfusions on mortality and hospital stay duration.

## 2. Materials and Methods

### 2.1. Study Design

This cross-sectional descriptive study was conducted in the ED of a tertiary care hospital. The annual number of ED visits is approximately 250,000. The study period covers patients admitted to the ED with a diagnosis of GI bleeding between 1 June 2021, and 1 June 2023. Only hospitalized patients who received RBCS transfusion were retrospectively analyzed. The primary outcomes were mortality rates and length of hospital stay among transfused patients.

### 2.2. Patient Selection

The inclusion criteria were as follows:

- Patients admitted to the ED with a diagnosis of GI bleeding and subsequently hospitalized;
- Patients who received RBCS transfusion;
- Patients aged 18 years and older.

The following exclusion criteria were applied:

- Patients under 18 years of age;
- Patients with a GI bleeding diagnosis followed on an outpatient basis;
- Patients diagnosed with GI bleeding who did not receive a transfusion;
- Patients who underwent cross-match testing for reasons other than GI bleeding;
- Patients with incomplete or inaccessible data.

**Figure 1:**
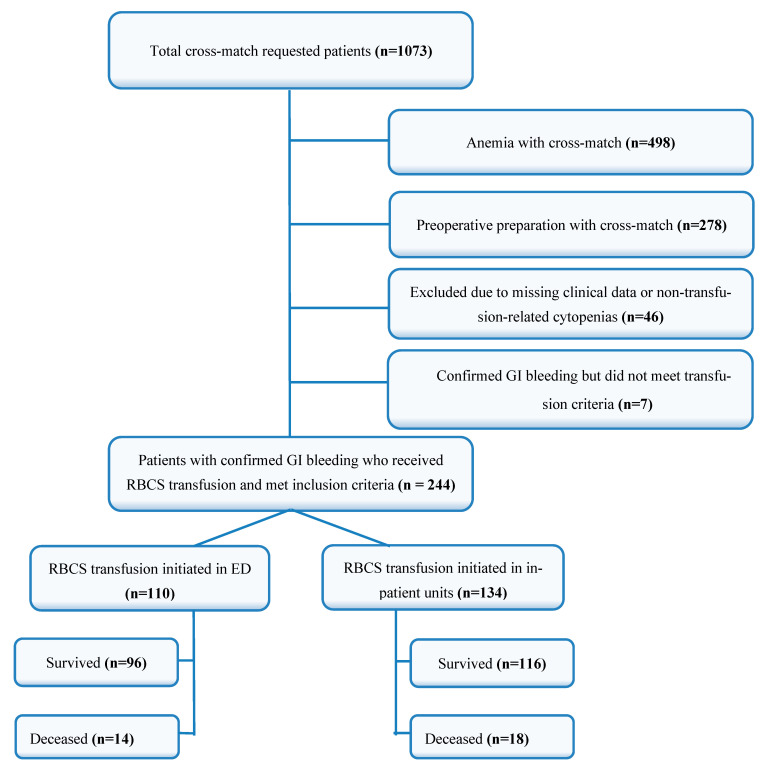
Flow chart of patient distribution and survival outcomes. RBCS: red blood cell suspension, GI: gastrointestinal.

After applying these criteria, a total of 244 patients were included in this study (Figure 1).

### 2.3. Data Collection

Data for this study were retrospectively obtained from the hospital information management system (HIMS) of the tertiary care hospital. Demographic data (age, gender), presenting complaints, vital signs, and laboratory parameters (Hb, hematocrit (Hct), blood urea nitrogen, creatinine, albumin, INR) were recorded. Additionally, information regarding the amount of RBCS transfused and the transfusion location (ED or inpatient unit) was collected. Outcome variables, including length of hospital stay and mortality status, were also retrieved from the same system and recorded for analysis.

### 2.4. Transfusion Protocol

In our institution, RBC transfusion for patients with GI bleeding is performed in accordance with a restrictive transfusion strategy, consistent with international guidelines such as those from the American College of Gastroenterology (ACG) and the European Society of Gastrointestinal Endoscopy (ESGE) [12,13]. According to this protocol, transfusion is generally initiated when Hb levels fall below 7 g/dL in hemodynamically stable patients without cardiovascular comorbidities, and below 8 g/dL in those with active bleeding or known cardiac disease [7,14]. Patients with persistent hemodynamic instability despite adequate fluid resuscitation may also receive transfusion regardless of initial Hb level, especially in the presence of clinical signs of ongoing hemorrhage [14]. This threshold-based approach has been adapted and standardized in our hospital practice to minimize the risk of rebleeding and other transfusion-related complications. The protocol also accounts for the clinical context, including comorbidities, severity of bleeding, and endoscopic findings, to guide individualized transfusion decisions.

In this study, “ED transfusion” was defined as any RBC transfusion that was initiated while the patient was physically located in the ED, regardless of whether the transfusion was completed there or continued in inpatient units. Upon arrival at the ED, patients with suspected GI bleeding are first evaluated by the emergency physician. Transfusion decisions in the ED are made by the attending physician based on institutional protocols and clinical presentation. Patients may then be admitted to inpatient units depending on severity, comorbidities, and endoscopic requirements.

### 2.5. Medication Management

Medication management followed standard institutional protocols. All patients received intravenous proton pump inhibitors upon admission. Anticoagulant or antiplatelet therapies were reviewed at presentation and temporarily withheld in cases of active bleeding. Resumption decisions were made individually based on hemostasis and comorbidity risk profiles, with cardiology or internal medicine consultation when indicated.

### 2.6. Statistical Analysis

Statistical analyses were performed using SPSS version 26.0 (IBM Corp., Armonk, NY, USA). Descriptive statistics are presented as means, standard deviations, medians, and interquartile ranges (IQRs) for continuous variables. Categorical variables were compared using the chi-square test. The Shapiro–Wilk test was used to assess the normality of distribution for continuous variables. For normally distributed data, the Mann–Whitney U test was applied. Variables that were statistically significant in the univariate analysis were included in the model. A total of eight variables were deemed potentially suitable for inclusion in a multivariate logistic regression analysis. Using a hierarchical block method, the contribution of each of the seven variables added to the model was evaluated using the Akaike Information Criterion (AIC), Bayesian Information Criterion (BIC), and Nagelkerke R^2^. It was determined that four of these variables did not contribute significantly to model fit.

All variables were assessed for multicollinearity through correlation analysis, Variance Inflation Factor (VIF), and tolerance values. Residuals and Cook’s Distance values were also examined. As no case had a Cook’s Distance > 1, no observations were excluded from the dataset. Ultimately, five variables were retained in the final model: MAP, albumin, creatinine, Hb, and length of hospital stay. The overall model fit was confirmed by the omnibus test (*p* < 0.01).

### 2.7. Ethical Approval

This study was approved by the Non-Interventional Clinical Research Ethics Committee of Kütahya Health Sciences University (approval number: 2023/09-29, dated 16 August 2023). Additionally, necessary permissions were obtained from the Kütahya Provincial Health Directorate Scientific Research Evaluation Committee (approval number: 2024/9, dated 31 January 2024).

## 3. Results

In the group receiving RBCS transfusion in the ED, 49 patients (44.5%) were male, and 61 (55.5%) were female. In contrast, among the patients who received transfusion in other hospital departments, 77 patients (57.7%) were male, and 57 (42.5%) were female. A statistically significant difference was observed between the groups regarding gender distribution (*p* = 0.045).

There was no statistically significant difference between the groups in terms of age (*p* = 0.074) or the presence of comorbid conditions such as coronary artery disease (CAD), diabetes mellitus (DM), hypertension (HT), stroke, atrial fibrillation (AF), chronic obstructive pulmonary disease (COPD), malignancy, cirrhosis, chronic kidney disease (CKD), and congestive heart failure (CHF). The demographic and comorbidity characteristics of the patients are summarized in Table 1.

The analysis of laboratory parameters showed that patients who received RBCS transfusion in the ED had significantly lower Hb and Hct levels compared to those in other hospital departments (*p* < 0.001). No statistically significant differences were observed between the groups in terms of other laboratory parameters, including blood urea nitrogen (BUN), creatinine, platelet count (Plt), international normalized ratio (INR), and albumin levels. The detailed laboratory values are presented in Table 2.

The comparison of hospital stays and mortality rates between the groups revealed that the median length of hospital stay was significantly shorter in the ED group (4 days) compared to other departments (5.5 days) (*p* < 0.001). The number of RBCS units transfused did not differ significantly between the groups (*p* = 0.142).

Regarding mortality outcomes, 14 patients (12.7%) in the ED group and 18 patients (13.4%) in the group that received transfusions in other departments were recorded as deceased. No statistically significant difference was found between the groups in terms of mortality rates (*p* = 0.871). The distribution of hospital stay duration and mortality rates is presented in Table 3.

**Figure 2:**
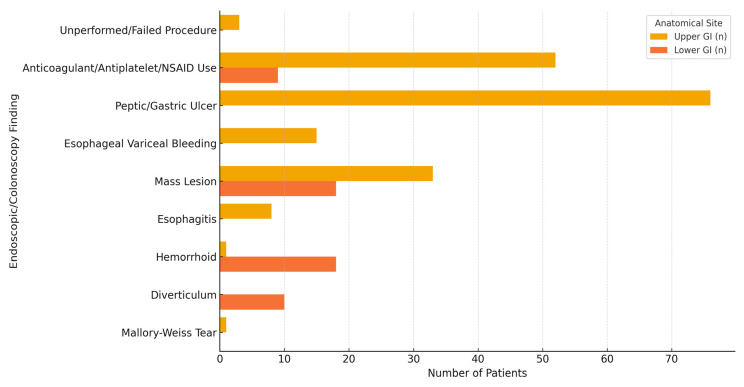
Distribution of endoscopic and colonoscopic findings according to the anatomical site of gastrointestinal bleeding. GI: gastrointestinal, NSAID: non-steroidal anti-inflammatory drug.

Among the total of 244 patients, 189 (77.5%) had UGIB and 55 (22.5%) had LGIB. A subgroup comparison between UGIB and LGIB patients revealed no statistically significant differences in terms of BUN, creatinine, Hct, platelet count, INR, albumin, length of hospital stay, or the number of RBCS units transfused (*p* > 0.05 for all). Although Hb levels were higher in UGIB patients compared to LGIB patients, the difference was not statistically significant (*p* = 0.085). Mortality rates were also comparable between groups (13.2% vs. 12.7%, *p* = 0.923) (Table 4). Endoscopic and colonoscopic evaluations revealed that peptic ulcers and variceal bleeding were the most common findings in upper GI bleeding, while hemorrhoids and diverticula predominated in lower GI bleeding (Figure 2).

Multivariate logistic regression analysis was performed to identify independent predictors of mortality among patients receiving RBCS transfusion. The model included five variables: mean arterial pressure (MAP), albumin, creatinine, Hb, and length of hospital stay, and transfusion location (ED vs. other departments). The model fit was confirmed using the omnibus test (*p* < 0.01), which indicated that the model was statistically significant. The model explained approximately 47% of the variance in mortality (Nagelkerke R^2^ = 0.472).

Among the five variables analyzed, all of them were identified as independent predictors of mortality (*p* < 0.05). The most significant predictors were MAP and albumin, both showing a protective effect, as indicated by OR values lower than 1. In contrast, Hb, creatinine, and length of hospital stay had OR values greater than 1, indicating an increased risk of mortality with higher values. The variable representing transfusion location (ED vs. other departments) did not reach statistical significance (*p* = 0.516), suggesting that the location of RBCS administration did not independently affect mortality. Therefore, the transfusion location was not included in the model. The detailed results of the logistic regression analysis are presented in Table 5.

**Figure 3:**
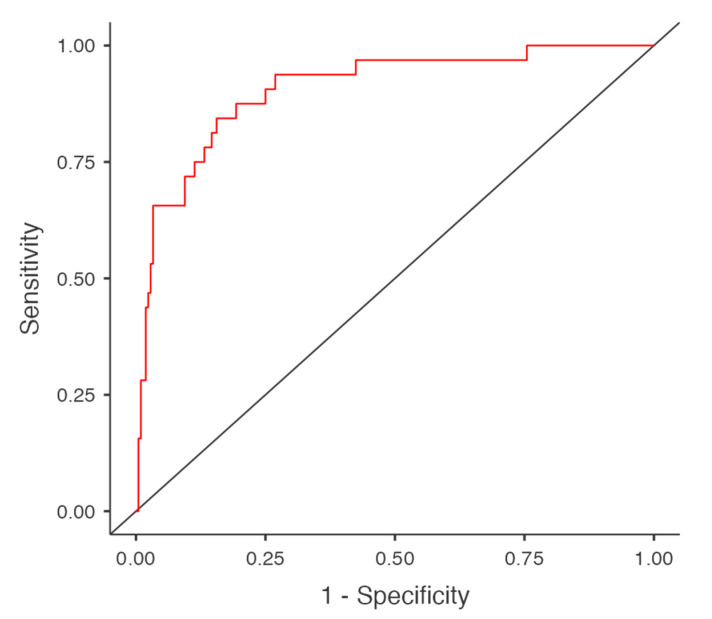
ROC curve of logistic regression model. The red line shows the model’s ROC curve; the black diagonal line is the reference line for random classification.

The diagnostic accuracy of the model was assessed using the area under the curve (AUC) of the receiver operating characteristic (ROC) curve, which was calculated as 0.906. The sensitivity and specificity were found to be 46.9% and 97.6%, respectively. The model’s accuracy was 91%, indicating good predictive power despite a moderate sensitivity (Figure 3).

## 4. Discussion

GI bleeding is a critical condition frequently encountered in EDs and remains a major cause of morbidity and mortality. Optimal transfusion strategies, particularly regarding the timing and location of RBCS administration, continue to be debated. Recent studies have shown that early transfusion in the ED can improve patient outcomes by stabilizing hemodynamics and reducing hospital stay without significantly increasing mortality [15,16].

In this study, we evaluated the effect of RBCS transfusion location on mortality and length of hospital stay in patients admitted with GI bleeding. Our findings revealed that transfusion performed in the ED was associated with a significantly shorter length of hospital stay, but with no difference in mortality compared to transfusion in inpatient units. These results suggest that the primary advantage of ED-based transfusion may be related to more efficient patient management rather than improved survival outcomes. Similar observations have been reported in recent guidelines, which emphasize a balance between early stabilization and careful transfusion practices to avoid complications [17]. However, due to the retrospective observational design, no causal inferences can be made regarding the impact of transfusion location on outcomes.

When evaluating the demographic and comorbidity characteristics of the patients, it was observed that male patients constituted the majority of the cohort. Similar studies have also demonstrated that male patients tend to present to EDs with GI bleeding more frequently [3]. For instance, a recent study by Arıkoğlu et al. (2025) [11] evaluated the effectiveness of various scoring systems, including GBS, AIMS65, Rockall, and the International Normalized Bleeding Score (INBS; ABC), in predicting hospital admission, transfusion need, and mortality. Consistent with our findings, they reported that approximately 65% of patients admitted to the ED due to GI bleeding were male [11].

However, our study revealed that patients requiring early RBCS transfusion and receiving it in the ED were predominantly female. This can be explained by the physiological differences in Hb levels and blood volume between genders. Since the threshold for RBCS transfusion is generally set at 7 g/dL, females, who typically have lower baseline Hb levels and reduced blood volume compared to males, may meet this criterion earlier. This observation is consistent with studies highlighting that lower baseline Hb levels in females may lead to earlier decisions for transfusion, even with moderate reductions [18,19].

In our study, comorbid conditions did not significantly influence the need for early RBCS transfusion in the ED. This finding is consistent with a 2024 study which reported that comorbidities such as antithrombotic therapy increased the risk of mortality and rebleeding in patients presenting with GI bleeding but did not directly affect transfusion requirements [4]. In contrast, other studies have suggested that advanced age and higher American Society of Anesthesiologists (ASA) scores may necessitate higher transfusion thresholds, indicating a more cautious approach [6].

Moreover, it has been proposed that patients with cirrhosis are more likely to require early transfusion due to factors such as decreased coagulation factors related to impaired liver function or variceal bleeding resulting from portal hypertension, both of which can lead to massive bleeding and increased mortality [20]. This variability in findings indicates that the relationship between comorbidities and transfusion requirements is not entirely clear, emphasizing the importance of individualized patient assessment. In our cohort, the lack of a significant association between comorbid conditions and early transfusion decisions suggests that clinical judgment primarily relies on acute clinical parameters and Hb levels rather than underlying chronic diseases.

When evaluating laboratory values, we observed that Hb and Htc levels were lower in patients receiving RBCS transfusion in the ED compared to those transfused in other units. This finding was expected, as restrictive transfusion strategies commonly used in GI bleeding recommend transfusion when Hb levels fall below 7 g/dL [21,22]. The lower Hb levels observed in the ED group are consistent with this guideline, indicating that our findings align with the current literature. Previous studies have reported that the BUN/creatinine ratio can be used as a potential biomarker to determine the localization and severity of GI bleeding. For instance, a study by Calim et al. (2025) demonstrated that patients with a BUN/creatinine ratio greater than 23.3 had higher rates of RBCS transfusion, endoscopic intervention, and mortality [23]. However, that study included both outpatient and inpatient cases. In contrast, our study exclusively analyzed hospitalized patients who received transfusion, which may explain why no significant differences in BUN, creatinine, platelet, INR, and albumin levels were observed between the groups.

Our study demonstrated that patients who received RBCS transfusion in the ED had a shorter length of hospital stay compared to those transfused in other inpatient units. This finding aligns with the concept that early transfusion in the ED may promote rapid hemodynamic stabilization and facilitate timely discharge. Similar results have been reported by Maher et al. (2020) and Chang et al. (2025), who found that empiric transfusion practices in the ED, driven by acute clinical parameters, can reduce the length of stay by addressing critical needs promptly, thereby optimizing hospital resource utilization [24,25]. However, this association should be interpreted with caution, as it may also reflect underlying differences in bleeding severity, clinical stability, or timing of presentation between ED and inpatient groups, rather than the transfusion setting itself. Additionally, the lack of detailed data regarding the interval between symptom onset, clinical deterioration, and transfusion initiation limits our ability to fully characterize the temporal dynamics influencing this association. Residual confounding by factors such as bleeding source, unmeasured comorbidities, and endoscopy timing may have influenced the observed associations.

Regarding mortality, our study found no statistically significant difference between the ED and inpatient transfusion groups, with in-hospital mortality rates of 12.7% and 13.4%, respectively. This outcome may reflect that patients transfused in the ED often present with more severe or acute hemodynamic compromise, and despite early intervention, underlying comorbidities and bleeding severity remain key drivers of mortality. Our ED group’s mortality was comparable to the 8.7% reported by Mark et al. (2020) in a large integrated health system cohort [9], suggesting that differences in institutional protocols, patient selection, and supportive care may influence outcomes. Additionally, Choi et al. (2025) showed that initiating transfusions within 4 h of presentation was associated with lower mortality (9.8% vs. 13.6%, *p* = 0.04) [15], emphasizing that early transfusion alone is not enough without careful patient selection and timely intervention. These findings underscore the need for integrated risk stratification, coordinated care pathways, and careful post-transfusion monitoring to improve patient outcomes and reduce mortality.

Our findings are also consistent with the guideline recommendations by the European Association for the Study of the Liver (2022), which emphasize that the decision for transfusion should not solely be based on Hb levels but should also consider the overall clinical status and comorbidities of the patient [26]. This approach helps prevent unnecessary transfusions and reduces the risk of transfusion-related complications while maintaining effective clinical outcomes. In line with this, our results align with current emergency and transfusion medicine guidelines (e.g., ESGE, ACG), which support restrictive transfusion strategies and recommend individualized patient management rather than fixed thresholds or universal protocols [13,21].

Among the cohort analyzed in this research, UGIB was more common than LGIB (77.5% vs. 22.5%), yet the two groups showed no statistically significant differences in laboratory parameters such as Hb, Hct, BUN, creatinine, or INR. Both groups required a median of 3 RBCS units, and hospital stay durations and mortality rates were also similar. These findings contrast with the common perception that UGIB presents more severely but are consistent with Kerbage et al. (2024), who emphasized that outcomes in GI bleeding are more closely linked to clinical status than bleeding location [4]. Similarly, Saydam et al. (2023) reported substantial overlap in severity and outcomes between UGIB and LGIB, especially among hospitalized patients [27]. The limited predictive accuracy of existing risk scores—highlighted by Radaelli et al. (2023) and further reflected in the structure of the Oakland score—supports the notion that anatomical classification alone is insufficient for reliable prognostication [28,29].

Our study identified five independent predictors of mortality: mean arterial pressure (MAP), albumin, length of hospital stay, Hb, and creatinine levels. Among these, MAP and albumin had the strongest protective associations, while transfusion location had no independent effect on mortality and was not included in the final model. This finding is consistent with the results of Chen et al. (2021), who demonstrated that low albumin levels were independently associated with increased mortality in patients presenting with acute UGIB, highlighting the prognostic value of albumin in critically ill patients [30].

Hb level also emerged as a significant predictor of mortality in our study. This aligns with the findings of Custovic et al. (2020) and Morarasu et al. (2023), who emphasized that Hb is a key variable in risk scoring systems like GBS and AIMS65, widely used for predicting outcomes in GI bleeding [31,32]. Lower Hb levels at admission have been linked to higher mortality risk, as also demonstrated by Aljarad et al. (2021), who found a moderate inverse relationship between Hb levels and mortality among hospitalized GI bleeding patients [33].

Additionally, creatinine levels were found to independently predict mortality in our cohort. This is in line with the study by Dajti et al. (2025), who reported that elevated creatinine was a significant predictor of in-hospital mortality among patients with lower GI bleeding [34]. Renal dysfunction in the context of acute bleeding may reflect systemic hypoperfusion and multi-organ failure, which could explain its association with poorer outcomes.

We also observed that prolonged hospital stay was correlated with increased mortality, likely reflecting the higher burden of comorbidities and advanced age in patients requiring extended care. Similar observations have been reported by Belete et al. (2024), who found that older patients with comorbid conditions tend to have longer hospital stays and higher mortality, underlining the importance of early risk stratification and multidisciplinary management [35].

## 5. Limitations

This study has several limitations that should be acknowledged. First, its retrospective design may have introduced selection and information bias, as data collection was based on medical records, which may contain inaccuracies. Additionally, our study was conducted at a single tertiary care center, which may limit the generalizability of the findings to other settings. Furthermore, the lack of precise data on transfusion timing, including the interval between symptom onset, hospital presentation and transfusion administration, and the absence of stratification based on the severity of bleeding and comorbid conditions may have influenced the results.

Moreover, since the decision to initiate transfusion in the ED versus inpatient units was based on real-time clinical judgment and institutional logistics, patients in these groups may differ systematically in disease severity and presentation characteristics. These differences could not be fully adjusted for, and thus, comparisons between the groups should be interpreted with caution. Selection bias remains a major limitation of this study, as the allocation of patients to ED or inpatient transfusion was not randomized and likely influenced by unmeasured clinical judgments and system-level factors. To validate our findings, prospective, multicenter studies with more comprehensive data collection are warranted.

## 6. Conclusions

Our study demonstrated that early RBCS transfusion performed in the ED is associated with a shorter length of hospital stay compared to transfusion in inpatient units. However, this difference did not result in a significant reduction in mortality. While this association may reflect more efficient patient management, it could also be influenced by differences in clinical severity, presentation timing, or institutional practices. Logistic regression analysis identified age, albumin levels, length of hospital stay, Hb levels, and creatinine levels as independent predictors of mortality, highlighting the importance of early risk stratification. These findings underscore the need for individualized transfusion strategies that consider both clinical status and patient-specific factors.

## Figures and Tables

**Table 1:** Comparison of Demographic and Comorbidity Profiles by Transfusion Location (ED vs. Inpatient Units).

Parameter		Total (n = 244)	ED (n = 110)	Other Departments (n = 134)	*p*-Value	Difference in Proportions (95% CI)
Age Median (IQR)		74 (20.3)	77 (23.8)	72 (17.8)	0.074	5 (−1 to 12)
Gender n (%)	Male	126 (51.6%)	49 (44.5%)	77 (57.5%)	0.045 *	0.128 (0.00406 to 0.252)
	Female	118 (48.4%)	61 (55.5%)	57 (42.5%)		
Comorbidity	CAD	76 (31.1%)	34 (30.9%)	42 (31.3%)	0.942	0.005 (−0.13 to 0.14)
	DM	63 (25.8%)	34 (30.9%)	29 (21.6%)	0.100	−0.120 (−0.26 to 0.02)
	HT	88 (36.1%)	44 (40%)	40 (32.8%)	0.246	−0.076 (−0.21 to 0.05)
	Stroke	14 (5.7%)	7 (6.4%)	7 (5.2%)	0.703	−0.05 (−0.32 to 0.29)
	AF	33 (13.5%)	19 (17.3%)	14 (10.4%)	0.121	−0.144 (−0.33 to 0.04)
	COPD	20 (8.2%)	5 (4.5%)	15 (11.2%)	0.060	0.219 (0.02 to 0.42)
	Malignancy	30 (12.3%)	15 (13.6%)	15 (11.2%)	0.563	−0.06 (−0.25 to 0.135)
	Cirrhosis	17 (7%)	6 (5.5%)	11 (82%)	0.400	0.105 (−0.13 to 0.34)
	CKD	13 (5.7%)	6 (5.5%)	8 (6.0%)	0.863	0.023 (−0.24 to 0.29)
	CHF	5 (2%)	4 (3.6%)	1 (0.7%)	0.113	−0.356 (−0.71 to 0.25)

The Mann–Whitney U test was used for continuous variables, and the chi-square test was used for categorical variables. The Hodges–Lehmann test was used for median differences. * Statistical significance level: *p* < 0.05. ED: emergency department; CAD: coronary artery disease; DM: diabetes mellitus; HT: hypertension; AF: atrial fibrillation; COPD: chronic obstructive pulmonary disease; CKD: chronic kidney disease; CHF: congestive heart failure; IQR: interquartile range.

**Table 2:** Comparison of Laboratory Parameters by Transfusion Location (ED vs. Inpatient Units).

Parameter	Total Median (IQR)	ED Median (IQR)	Other Departments Median (IQR)	*p*-Value	Median Difference (%95 CI)
BUN	31 (32)	34 (34)	30 (28)	0.742	4 (−4 to 6)
Creatinine (mg/dL)	1.12 (0.75)	1.15 (0.825)	1.10 (0.635)	0.647	0.05 (−0.09 to 0.16)
Hemoglobin (g/dL)	6.70 (3.125)	6.05 (2.175)	7.50 (4.075)	<0.001 *	−1.45 (−2.3 to −1.01)
Hematocrit (%)	22.45 (9.25)	20.40 (6.825)	24.05 (10.90)	<0.001 *	−3.25 (−6.3 to −2.4)
Platelet (10^9^;/L)	249 (143)	260 (145.75)	244 (138.25)	0.789	−11 (−31 to 15)
INR	1.17 (0.423)	1.18 (0.480)	1.15 (0.377)	0.958	0.03 (−0.06 to 0.06)
Albumin (g/L)	32 (8)	32 (9.45)	32 (8)	0.580	0.0 (−2 to 1)

The Mann–Whitney U test was used for continuous variables. The Hodges–Lehmann test was used for median differences. * Statistical significance level: *p* < 0.05. BUN: blood urea nitrogen; INR: international normalized ratio; ED: emergency department; IQR: interquartile range.

**Table 3:** Comparison of Length of Hospital Stay, Transfusion Amount, and Mortality Between Patients Transfused in the Emergency Department and Inpatient Units.

Parameter	Total Median (IQR)	ED Median (IQR)	Other Departments Median (IQR)	*p*-Value	Median Differences (%95 CI)
Length of Stay (days)	5 (5)	4 (7)	5.5 (5)	<0.001 *	−1.5 (−3 to −0.5)
RBCS Units Transfused	3 (2)	3 (2)	3 (1)	0.142	0 (0 to 1.0)
Survived n (%)	212 (86.9%)	96 (87.3%)	116 (86.6%)	0.871	0.01 (−0.2 to 0.17)
Deceased n (%)	32 (13.1%)	14 (12.7%)	18 (13.4%)		

The Mann–Whitney U test was used for continuous variables, and the chi-square test was used for categorical variables. The Hodges–Lehmann test was used for median differences. * Statistical significance level: *p* < 0.05. ED: emergency department; RBCS: red blood cell suspension; IQR: interquartile range.

**Table 4:** Comparison of laboratory and clinical parameters between UGIB and LGIB patients.

Parameter	Total Median (IQR)	UGIB Median (IQR)	LGIB Median (IQR)	*p*-Value	Median Difference (%95 CI)
BUN	31 (32)	34 (33)	28 (31.5)	0.296	6 (−4.5 to 14.8)
Creatinine (mg/dL)	1.12 (0.75)	1.12 (0.70)	1.10 (0.925)	0.724	0.02 (−0.34 to 0.24)
Hemoglobin (g/dL)	6.70 (3.125)	6.80 (2.175)	6.40 (4.075)	0.085	0.40 (−0.10 to 1.56)
Hematocrit (%)	22.45 (9.25)	22.50 (9.80)	21.6 (7.45)	0.137	0.9 (−0.56 to 4.09)
Platelet (10^9^/L)	249 (143)	247 (144)	223 (139)	0.788	24 (−39.6 to 30.104)
INR	1.17 (0.423)	1.18 (0.430)	1.12 (0.385)	0.510	0.06 (−0.46 to 0.94)
Albumin (g/L)	32 (8)	32 (8)	33 (9.50)	0.539	−1 (−2.48 to 1.30)
Length of Stay (days)	5 (5)	5 (5)	5 (6.50)	0.480	0 (−1 to 2)
RBCS Units Transfused	3 (2)	3 (2)	3 (1.50)	0.692	0 (−1.5 to 0.5)
Survived n (%)	212 (86.9%)	164 (86.8%)	48 (87.3%)	0.923	0.007 (−0.162 to 0.147)
Deceased n (%)	32 (13.1%)	25 (13.2%)	7 (12.7%)		

The Mann–Whitney U test was used for continuous variables and the chi-square test was used for categorical variables. The Hodges–Lehmann test was used for median differences. BUN: blood urea nitrogen; INR: international normalized ratio; RBCS: red blood cell suspension; IQR: interquartile range; UGIB: Upper gastrointestinal bleeding; LGIB: Lower gastrointestinal bleeding.

**Table 5:** Multivariate logistic regression analysis of mortality.

Variables	Estimate (b)	Wald	*p*-Value	Odds Ratio (OR)	OR 95% CI
Intercept	4.8489	2.82	0.005	127.600	4.407–3694.828
MAP (mmHg)	−0.0841	−3.82	<0.001	0.919	0.88–0.96
Albumin (g/L)	−0.1494	−3.80	<0.001	0.861	0.80–0.93
Length of Hospital Stay	0.0676	2.76	0.006	1.070	1.020–1.123
Hemoglobin (g/dL)	0.2890	3.22	0.001	1.335	1.12–1.59
Creatinine (mg/dL)	0.4489	2.67	0.008	1.57	1.12–2.18

MAP: mean arterial pressure; OR: odds ratio; CI: confidence interval; Statistical significance level: *p* < 0.05.

## Data Availability

The datasets used and/or analyzed during the current study are available from the corresponding author on reasonable request.

## Abbreviations

ABC	Age, Blood Tests, and Comorbidities
ACG	American College of Gastroenterology
AF	Atrial Fibrillation
AIMS65	Albumin–International Normalized Ratio–Mental Status–Systolic Blood Pressure-65
ASA	American Society of Anesthesiologists
BUN	Blood Urea Nitrogen
CAD	Coronary Artery Disease
CHF	Congestive Heart Failure
CKD	Chronic Kidney Disease
COPD	Chronic Obstructive Pulmonary Disease
ED	Emergency Department
ES	Erythrocyte Suspension
ESGE	European Society of Gastrointestinal Endoscopy
GI	Gastrointestinal
GBS	Glasgow–Blatchford Score
Hb	Hemoglobin
Hct	Hematocrit
HT	Hypertension
IQR	Interquartile Range
INBS	International Normalized Bleeding Score
INR	International Normalized Ratio
MAP	Mean Arterial Pressure
NV-AUGIB	Non-Variceal Acute Upper Gastrointestinal Bleeding
OR	Odds Ratio
Plt	Platelet
RBC	Red Blood Cell
RBCS	Red Blood Cell Suspension
ROC	Receiver Operating Characteristic
UGIB	Upper Gastrointestinal Bleeding
LGIB	Lower Gastrointestinal Bleeding
SPSS	Statistical Package for the Social Sciences
